# Stereoselective synthesis of tetrasubstituted alkenes via a sequential carbocupration and a new sulfur–lithium exchange

**DOI:** 10.3762/bjoc.8.248

**Published:** 2012-12-18

**Authors:** Andreas Unsinn, Cora Dunst, Paul Knochel

**Affiliations:** 1 Department Chemie, Ludwig-Maximilians-Universität München, Butenandtstr. 5-13, 81377 München, Germany

**Keywords:** alkenes, carbometalation, copper, regioselectivity, stereoselectivity

## Abstract

We have designed a new sequential carbocupration and sulfur–lithium exchange that leads stereo- and regioselectively to trisubstituted alkenyllithiums. Subsequent trapping with various electrophiles yields tetrasubstituted olefins with good control of the double-bond geometry (*E*/*Z* ratio up to 99:1). The novel sulfur–lithium exchange could be extended to the stereoselective preparation of *Z*-styryl lithium derivatives with almost complete retention of the double-bond geometry.

## Introduction

The stereoselective synthesis of tetrasubstituted alkenes is an important synthetic goal, which may be achieved by carbometalation methods [1–9]. The Normant carbocupration of terminal acetylenes allows the stereoselective preparation of trisubstituted alkenes with excellent *E*/*Z* ratio [10–12]. However, in order to obtain tetrasubstituted alkenes, a carbometalation of an internal alkene is required. This reaction is usually difficult due to steric hindrance and proceeds only if electron-withdrawing groups are attached to the alkyne unit to facilitate the carbometalation step. Recently, we studied the chemistry of alkenyl sulfides and their use for carbometalation extensively [13].

**Scheme 1:**
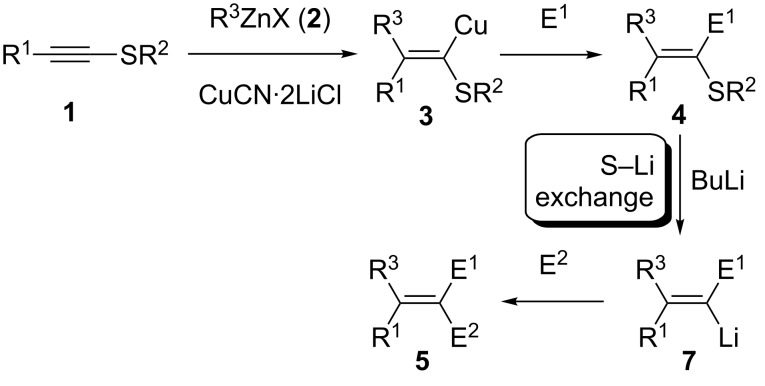
Synthesis of tetrasubstituted olefins by a successive carbocupration and S–Li exchange.

Therefore, we envisioned using an alkynyl thioether such as **1** as an activated alkyne. After a carbocupration of the alkynyl thioether **1** with the organozinc reagent **2** in the presence of CuCN·2LiCl [14], the alkenylcopper species **3** should be obtained. Stereoselective quenching with an electrophile (E^1^) should afford the tetrasubstituted alkenyl thioether **4**. Extensive experimentation showed that thioethers **4** do not undergo Ni- or Pd-catalyzed cross couplings leading to products of type **5** (R = Me, Ph) [15–16]. Thus, we designed a new sulfur–lithium exchange (Scheme 1).

**Scheme 2:**
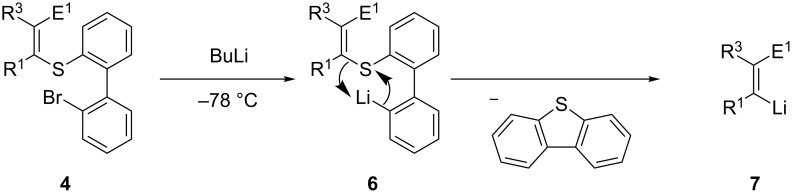
Proposed mechanism of the sulfur–lithium exchange starting with the alkenyl thioether **4**.

Sulfur–lithium exchanges proceed only readily with sulfoxides [17–19] and these reactions are often complicated by radical side reactions [20–21]. This new, direct sulfur–lithium exchange on an alkenyl thioether of type **4** involves the use of a bromobiphenyl R-group, which by treatment with BuLi at low temperatures, undergoes first a fast bromine–lithium exchange leading to an intermediate biphenyllithium derivative of type **6**, followed by an intramolecular ring-closing sulfur–lithium exchange [22] leading to the desired alkenyllithium **7** (Scheme 2).

Subsequent quenching with a different electrophile E^2^ should afford the tetrasubstituted alkene of type **5**; (Scheme 1). Herein, we demonstrate the feasibility of this methodology and thus prepare tetrasubstituted alkenes with *E*/*Z* stereoselectivities up to 99:1. Furthermore, we show that this sulfur–lithium exchange can be extended to the stereoselective preparation of *Z*-styryl derivatives.

## Results and Discussion

**Scheme 3:**
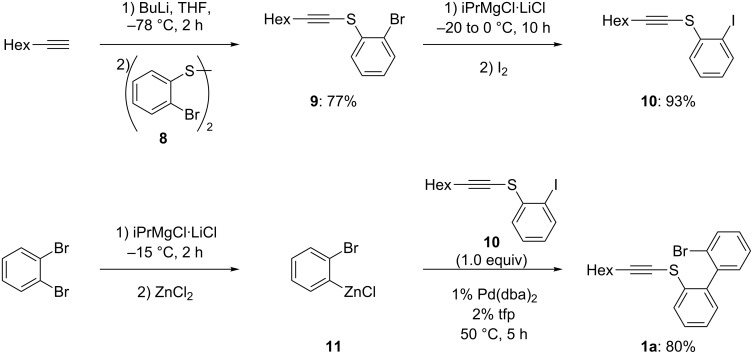
Synthesis of the precursor **1a**.

First, we wish to report the synthesis of the alkynyl biphenyl thioether **1a** required for the carbometalation step. Thus, octyne was deprotonated with butyllithium (1.1 equiv, THF, −78 °C, 2 h) followed by the addition of the diaryl disulfide [23] (**8**: 1.1 equiv, −78 °C to 25 °C, 3 h) providing the bromothioether **9** in 77% yield. Direct Pd-catalyzed Negishi cross-coupling [24–28] of **9** with an arylzinc derivative failed. However, the bromide **9** could be readily converted to the corresponding iodide **10** by a bromine–magnesium exchange using iPrMgCl·LiCl [29–35] followed by iodolysis leading to the iodide **10** in 93% yield. Treatment of 1,2-dibromobenzene with iPrMgCl·LiCl at −15 °C for 2 h followed by a transmetalation with ZnCl_2_ gives the required zinc reagent **11**, which undergoes a Negishi cross-coupling with the iodide **10** at 50 °C (5 h) leading to the alkynyl thioether **1a** in 80% yield (Scheme 3).

**Scheme 4:**
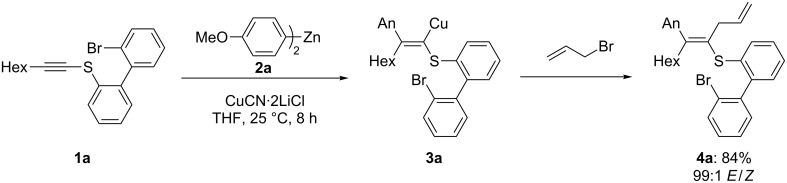
Carbocupration of the thioether **1a** leading to the tetrasubstituted alkene **4a**.

The harsh cross-coupling conditions may be due booth to the presence of the ortho-bromo substitution in the zinc reagent **11**, which considerably reduces the nucleophilicity of this arylzinc reagent by inductive effects, and also to the sulfur atom of the electrophile, which poisons the Pd catalyst. With the thioether **1a** in hand, we have performed the Normant carbocupration with di-*para*-anisylzinc (An_2_Zn: **2a**) according to a procedure previously developed by us [36]. Thus, the reaction of **1a** (1.0 equiv) with An_2_Zn (1.5 equiv, THF) in the presence of CuCN·2LiCl (1.5 equiv) at 25 °C for 8 h produces the intermediate copper reagent **3a**, which, after allylation with allyl bromide, provides the thioether **4a** in 84% yield and an *E*/*Z* ratio of 99:1 (Scheme 4). The reaction of **3a** with other typical electrophiles is possible, but proceeds in moderate yields due to the low reactivity of copper reagent **3a**.

**Scheme 5:**
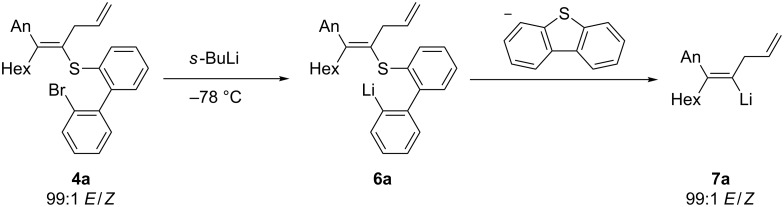
Synthesis of the alkenyllithium reagent **7a** by an S–Li exchange.

The bromothioether **4a** was then treated with *s-*BuLi (1.3 equiv, −78 °C, 10 min), leading to the formation of the intermediate aryllithium **6a**, which undergoes the desired intramolecular sulfur–lithium exchange affording the alkenyllithium reagent **7a** (Scheme 5).

**Scheme 6:**
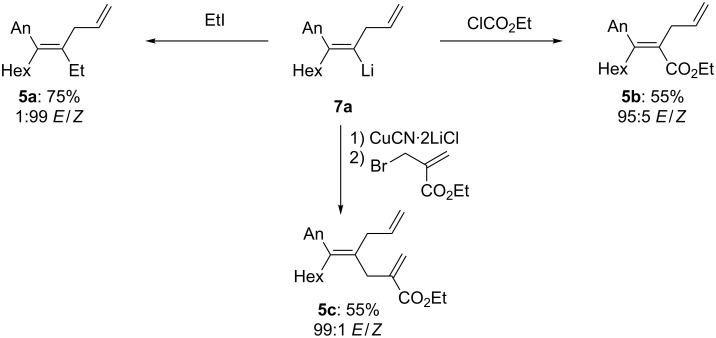
Quenching of the alkynyllithium **7a**. (Product ratios and diastereoselectivities were determined by ^1^H- and 2D-NMR.)

This alkenyllithium was quenched with typical electrophiles with a high retention of the double-bond geometry. Thus, the treatment of **7a** with EtI (2 equiv, −78 °C, 15 min) provides the tetrasubstituted alkene **5a** in 75% yield and an *E*/*Z* ratio of 1:99. Direct carboxylation by the reaction with ethyl chloroformate (1.1 equiv, −78 °C, 15 min) furnishes the corresponding unsaturated ethylester **5b** in 55% isolated yield and an *E*/*Z* ratio of 95:5. Finally, a copper-catalyzed allylation with ethyl 2-(bromomethyl)acrylate [37] (1.5 equiv, −78 to 0 °C, 2 h) affords the triene **5c** in 55% yield and an *E*/*Z* ratio of 99:1 (Scheme 6).

These quenching experiments demonstrate that this new method based on a successive carbocupration and sulfur–lithium exchange allows the stereoselective preparation of various tetrasubstituted alkenes. Since Normant has shown that various alkylcopper species add to alkynyl thioethers [38–40], the use of a bromobiphenyl substituent (R^2^) on the sulfur may allow a general stereoselective synthesis of tetra-substituted alkenes.

**Scheme 7:**
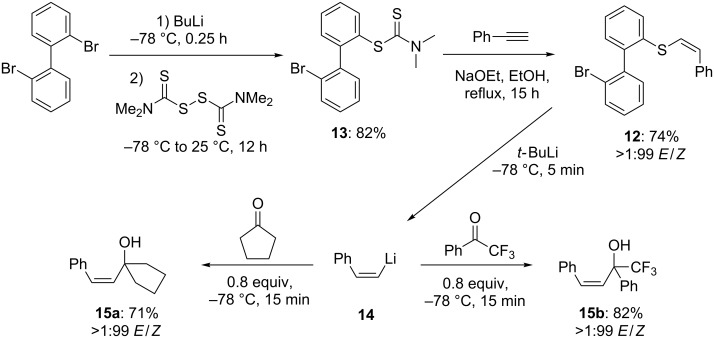
Synthesis and quenching of *Z*-styryllithium.

In order to prove that this new sulfur–lithium exchange has further applications in the stereoselective synthesis of alkenes, we prepared the *Z*-alkenyl thioether **12** starting from 2,2’-dibromobiphenyl. Thus, the performance of a double bromine–lithium exchange with BuLi (1.1 equiv, −78 °C, 0.25 h) followed by a quenching with tetramethylthiuram disulfide (1.1 equiv, −78 to 25 °C, 12 h) furnishes the dithiocarbamate **13** in 82% yield. Since the reduction to the free thiol is hard to achieve due to dibenzothiophene formation [41], we performed an in situ deprotection and stereoselective addition to phenylacetylene [42] (1.5 equiv, 1.25 equiv NaOEt, EtOH, reflux, 15 h) yielding the *Z*-alkenyl thioether **12** in 74% yield (Scheme 7).

Treatment of **12** with *t*-BuLi (1.6 equiv, −78 °C, 10 min) provides directly the *Z*-styryllithium **14**, which stereoselectively adds to α,α,α-trifluoroacetophenone (0.8 equiv, −78 °C, 0.5 h) and cyclopentanone (0.8 equiv, −78 °C, 0.5 h) to afford the expected tertiary allylic alcohols **15a**–**b** in 71–82% yield and *E*/*Z* ratios of >1:99.

## Conclusion

In summary, we have reported tetrasubstituted olefins with excellent *E*/*Z* ratios using a sequential carbocupration and a new sulfur–lithium exchange involving an alkenyl thioether bearing a 2’-bromobiphenyl substituent, which triggers efficiently the sulfur–lithium exchange. Extension to the stereoselective preparation of Z-styryllithium was shown.

## Supporting Information

File 1 Experimental details and characterization data of new compounds.
