# Supplementary material for: Stereoselective synthesis of tetrasubstituted alkenes via a sequential carbocupration and a new sulfur–lithium exchange
Source: Beilstein J Org Chem. 2012 Dec 18;8:2202–6. doi: 10.3762/bjoc.8.248 (PMC3556915; doi:10.3762/bjoc.8.248)
Supplement: File 1 — Experimental details and characterization data of new compounds. [file Beilstein_J_Org_Chem-08-2202-s001.pdf]

Supporting Information

for

Stereoselective synthesis of tetra-substituted alkenes via a sequential carbocupration and a new sulfur–lithium exchange

Andreas Unsinn, Cora Dunst and Paul Knochel*

Address: Department Chemie, Ludwig-Maximilians-Universität München, Butenandtstr. 5-13, 81377 München, Germany

Email: Paul Knochel* - paul.knochel@cup.uni-muenchen.de

* Corresponding author

Experimental details and characterization data of new compounds

General

All reactions were carried out under an argon atmosphere in flame-dried glassware. Syringes that were used to transfer anhydrous solvents or reagents were purged with argon prior to use. THF was continuously heated under reflux and freshly distilled from sodium benzophenone ketyl under nitrogen. Yields refer to isolated yields of compounds estimated to be >95 % pure as determined by ^{1}H NMR (25 °C) and capillary GC. NMR spectra were recorded on solutions in deuterated chloroform ($CDCl_3$) with residual chloroform (δ 7.25 ppm for ^{1}H NMR and δ 77.0 ppm for ^{13}C NMR) or d_6-DMSO (δ 2.49 ppm for ^{1}H NMR and δ 39.5 ppm for ^{13}C NMR). Column chromatographical purifications were performed by using SiO_2 (0.040–0.063 mm, 230–400 mesh ASTM) from Merck if not indicated otherwise.

Typical procedure for the carbocupration of alkynyl sulfides with functionalized diorganozinc reagents (TP 1)

In a similar way as described in [1], a dry and argon-flushed Schlenk flask, equipped with a magnetic stirring bar and a septum, was charged with the diorganozinc reagent of type $R_2Zn{\cdot}2MgX_2{\cdot}LiCl$ (1.5 equiv) and cooled to −20 C. CuCN·2LiCl (1.5 equiv) was dropwise added and the resulting mixture was stirred for 30 min. Then, the alkynyl sulfide was added, warmed to 25 °C and stirred for the indicated time. The carbocupration progress was monitored by GC analysis of the reaction aliquots, which where quenched with a mixture of sat. aq. NH_4Cl/NH_3 (25% in H_2O) = 9:1 with tetradecane as internal standard.

Typical procedure for the sulfur–lithium exchange (TP 2)

In a dry and argon-flushed Schlenk flask equipped with a septum and a magnetic stirring bar 2’-bromobiphenyl thioether (1 mmol) was dissolved in THF (10 mL) and the solution cooled to −78 °C. Then the organolithium was added and the reaction mixture was stirred for 10 min.

Synthesis of 2'-bromobiphenyl-2-yl oct-1-yn-1-yl sulfide (1a)

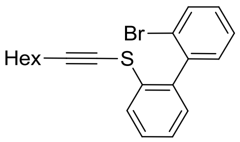

A: Synthesis of 1,1'-disulfanediylbis(2-bromobenzene) (8)

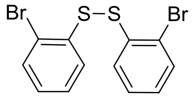

This compound was prepared from commercially available 2-bromothiophenole according to the procedure reported by Wilson and Tarbell [2].

B: Synthesis of 1-bromo-2-(oct-1-yn-1-ylsulfanyl)benzene (9)

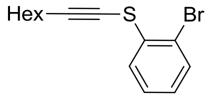

A dry and argon-flushed Schlenk flask, equipped with a magnetic stirring bar and a septum, was charged with 1-octyne (1.1 g, 10 mmol) in THF (10 mL). *n*BuLi (4.4 mL, 11 mmol) was slowly added at −78 °C and the resulting solution was stirred for 2 h. Then, 1,1'-disulfanediylbis(2-bromobenzene) (**8**) (4.1 g, 11 mmol) was added at this temperature and the resulting mixture was stirred for 3 h while warming up to 25 °C. The reaction mixture was quenched with sat. Na_2CO_3 (100 mL) and the resulting mixture was extracted with diethyl ether (3 × 100 mL). The combined organic layers were dried ($MgSO_4$), filtered, and concentrated under reduced pressure. Purification by flash chromatography (pentane) yielded 1-bromo-2-(oct-1-yn-1-ylsulfanyl)benzene (**9**, 1.63 g, 77%) as a yellow oil.

^{1}H NMR (DMSO, 400 MHz) *δ* (ppm): 7.65–7.62 (m, 2H), 7.50–7.46 (m, 1H), 7.23–7.19 (m, 1H), 2.51 (t, *J* = 6.9 Hz, 2H), 1.58–1.50 (m, 2H), 1.42–1.35 (m, 2H), 1.26–1.27 (m, 4H), 0.87–0.83 (m, 3H).

^{13}C NMR (DMSO, 100 MHz) *δ* (ppm): 134.0, 132.8, 128.8, 128.0, 126.2, 118.4, 102.9, 63.3, 30.7, 28.0, 27.9, 22.0, 19.5, 13.9.

IR (Diamond-ATR, neat) $\tilde{\nu}$ (cm^{-1}): 2954, 2928, 2856, 1575, 1446, 1428, 1255, 1104, 1036, 1018, 743, 726, 710.

MS (70 eV, EI) *m/z* (%): 298 (50), 296 (M^+, 45), 229 (20), 227 (31), 225 (11), 190 (14), 188 (25), 188 (14), 183 (25), 181 (26), 175 (16), 174 (42), 173 (19), 160 (14), 149 (17), 148 (95), 147 (100), 146 (20), 145 (13), 141 (27), 115 (12), 109 (51), 108 (20), 108 (14), 107 (17), 102 (32), 93 (14), 81 (14), 79 (33), 71 (23), 69 (12), 67 (70), 55 (13), 44 (10), 44 (15).

HRMS (EI) for **$C_{14}H_{17}BrS$**: (296.0234): 296.0225.

C: Synthesis of 1-iodo-2-(oct-1-yn-1-ylsulfanyl)benzene (10)

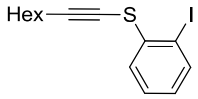

A dry and argon-flushed Schlenk flask, equipped with a magnetic stirring bar and a septum, was charged with 1-bromo-2-(oct-1-yn-1-ylsulfanyl)benzene (**9**, 2.1 g, 10 mmol) and cooled to −20 °C. iPrMgCl·LiCl (17 mL, 11 mmol) was added at −20 °C

and the resulting mixture was stirred for 10 h warming slowly to 0 °C. Then, a solution of I_2 (5.6 g, 22 mmol) in THF (20 mL) was added and stirred at this temperature for 15 min. The reaction mixture was quenched with sat. $Na_2S_2O_3$ (100 mL) and the resulting mixture was extracted with diethyl ether (3 × 100 mL). The combined organic layer was dried ($MgSO_4$), filtered, and concentrated under reduced pressure. Purification by flash chromatography (pentane + 2 vol % NEt_3) yielded 1-iodo-2-(oct-1-yn-1-ylsulfanyl)benzene (**10**, 2.41 g, 93%) as a yellow oil.

^{1}H NMR (DMSO, 400 MHz) *δ* (ppm): 7.81 (dd, *J* = 7.8 Hz, 1H), 7.63–7.60 (m, 1H), 7.52–7.47 (m, 1H), 7.04–7.00 (m, 1H), 2.51–2.48 (m, 2H), 1.57–1.50 (m, 2H), 1.42–1.35 (m, 2H), 1.30–1.25 (m, 4H), 0.87–0.83 (m, 3H).

^{13}C NMR (DMSO, 100 MHz) *δ* (ppm): 139.2, 137.6, 129.3, 127.9, 125.6, 102.6, 94.0, 65.0, 30.7, 28.0, 27.9, 22.0, 19.5, 13.9.

IR (Diamond-ATR, neat) $\tilde{\nu}$ (cm^{-1}): 2954, 2926, 2856, 1568, 1558, 1440, 1424, 1378, 1324, 1254, 1094, 1036, 1008, 938, 742, 702, 644.

MS (70 eV, EI) *m/z* (%): 344 (M^+, 90), 275 (23), 273 (20), 236 (39), 174 (15), 173 (17), 148 (43), 147 (100), 146 (31), 141 (25), 128 (13), 109 (47), 109 (16), 108 (17), 108 (19), 102 (22), 81 (15), 79 (27), 71 (16), 69 (13), 67 (58), 57 (14), 55 (19), 43 (13), 41 (17).

HRMS (EI) for **$C_{14}H_{17}IS$**: (344.0096) 344.0101.

D: Synthesis of 2'-bromobiphenyl-2-yl oct-1-yn-1-yl sulfide (1a)

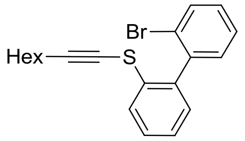

A dry and argon-flushed Schlenk flask, equipped with a magnetic stirring bar and a septum, was charged with iPrMgCl·LiCl (9.4 mL, 12.6 mmol) and cooled to −20 °C. 1,2-Dibromobenzene (2.8 g, 12 mmol) was slowly added at this temperature and stirred at −15 °C for 2 h. Then, $ZnCl_2$ (12.6 mL, 12.6 mmol, 1 M in THF) was added and the resulting mixture was stirred at this temperature for 20 min. The resulting solution was cannulated to a new Schlenk flask equipped with 1-iodo-2-(oct-1-yn-1-ylsulfanyl)benzene (**10**, 2.8 g, 12 mmol, 1 m in THF), $Pd(dba)_2$ (115 mg, 1 mol %) and P(*o*-furyl)$_3$ (93 mg, 2 mol %) and stirred at 50 °C for 5 h. The reaction mixture was then quenched with sat. NH_4Cl (100 mL) and the resulting mixture was extracted

with diethyl ether (3 × 100 mL). The combined organic layers were dried over $MgSO_4$ and concentrated in vacuo. The crude residue was purified by flash column chromatography (pentane + 2 vol % NEt_3) to give **1a** (2.3 g, 80%) as a yellow oil.

^{1}H NMR ($CDCl_3$, 300 MHz) *δ* (ppm): 7.82–7.79 (m, 1H), 7.71–7.67 (m, 1H), 7.47–7.35 (m, 2H), 7.31–7.24 (m, 3H), 7.16–7.13 (m, 1H), 2.24 (t, *J* = 6.9 Hz, 2H), 1.65–1.56 (m, 2H), 1.50–1.40 (m, 2H), 1.36–1.29 (m, 4H), 0.94–0.89 (m, 3H).

^{13}C NMR ($CDCl_3$, 75 MHz) *δ* (ppm): 139.8, 138.4, 133.5, 132.8, 131.2, 129.8, 129.7, 128.8, 127.3, 125.8, 125.7, 123.8, 100.6, 64.6, 31.3, 28.6, 22.5, 20.3, 14.0.

IR (Diamond-ATR, neat) $\tilde{\nu}$ (cm^{-1}): 3052, 2952, 2926, 2855, 1582, 1561, 1453, 1434, 1421, 1377, 1324, 1159, 1118, 1078, 1052, 1035, 1027, 1002, 942, 748, 729, 686, 658.

MS (70 eV, EI) *m/z* (%): 372 (M^+, 5), 294 (20), 293 (100), 221 (11), 184 (21).

HRMS (EI) for **$C_{20}H_{21}BrS$** (372.0547) 372.0539.

Synthesis of 2-(((1*E*)-1-allyl-2-(4-methoxyphenyl)oct-1-en-1-yl)thio)-2'-bromobiphenyl (4a)

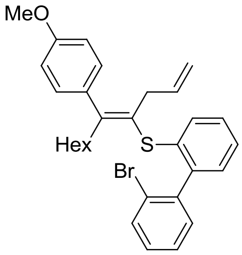

Prepared according to TP 1 from 2'-bromobiphenyl-2-yl oct-1-yn-1-yl sulfide (**1a**) (373 mg, 1 mmol) and bis(4-methoxyphenyl)zinc [3] (**2a**, 4 mL, 1.5 mmol) [carbometalation conditions: 25 °C, 8 h]. Then, the reaction mixture was cooled to −40 °C and allyl bromide (0.29 mL, 3 mmol) was added. The solution was stirred for 30 min at this temperature followed by 30 min at 0 °C. The reaction mixture was quenched with sat. NH_4Cl/NH_3 (25% in H_2O) = 9:1 (100 mL) and the resulting mixture was extracted with diethyl ether (3 × 100 mL). The combined organic layer was dried ($MgSO_4$), filtered, and concentrated under reduced pressure. Purification by flash chromatography (pentane + 2 vol % NEt_3) yielded **4a** (367 mg, 84%, *E/Z* = 99:1) as a yellow oil.

^{1}H NMR ($CDCl_3$, 300 MHz) *δ* (ppm): 7.68 (d, *J* = 7.9 Hz, 1H), 7.41–7.16 (m, 7H), 7.00 (d, *J* = 8.5 Hz, 2H), 6.85 (d, *J* = 8.7 Hz, 2H), 5.79–5.65 (m, 1H), 4.90–4.75 (m, 2H),

3.80 (s, 3H), 2.71 (d, *J* = 6.1 Hz, 2H), 2.46 (d, *J* = 4.1 Hz, 2H), 1.28–1.15 (m, 8H), 0.81 (t, *J* = 6.5 Hz, 3H).

^{13}C NMR ($CDCl_3$, 75 MHz) δ (ppm): 158.4, 149.6, 141.9, 141.4, 136.6, 136.5, 135.1, 134.2, 132.6, 131.5, 130.4, 129.8, 129.1, 129.0, 128.3, 127.5, 126.9, 125.8, 123.9, 115.4, 113.4, 107.5, 55.2, 38.3, 37.1, 31.6, 29.0, 28.1, 22.5, 14.1.

IR (Diamond-ATR, neat) $\tilde{\nu}$ (cm^{-1}): 2952, 2926, 2855, 1582, 1561, 1453, 1434, 1421, 1377, 1323, 1159, 1118, 1078, 1052, 1027, 1002, 942, 863, 748, 729, 686, 658.

MS (70 eV, EI) *m/z* (%): 523 (26), 522 (78), 521 (26), 520 (M^+, 70), 442 (24), 441 (65), 216 (10), 215 (55), 187 (35), 186 (18), 185 (19), 184 (15), 174 (14), 173 (87), 171 (11), 161 (46), 159 (23), 158 (12), 147 (20), 145 (13), 121 (100).

HRMS (EI) for **$C_{30}H_{33}BrOS$**: (520.1435) 520.1432.

Synthesis of 1-[(1*Z*)-2-ethyl-1-hexylpenta-1,4-dien-1-yl]-4-methoxybenzene (5a)

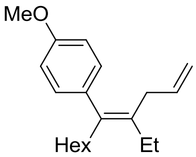

Prepared according to TP 2 from 2-(((1*E*)-1-allyl-2-(4-methoxyphenyl)oct-1-en-1-yl)thio)-2'-bromobiphenyl (**4a**, 436 mg, 1 mmol) and *s*-BuLi (1.35 mL, 1.1 mmol). After 10 min iodethane (312 mg, 2 mmol) was added and the solution was stirred for 15 min. The reaction mixture was quenched with sat. NH_4Cl sol. (25 mL) and the resulting mixture was extracted with diethyl ether (3 × 50 mL). The combined organic layer was dried ($MgSO_4$), filtered, and concentrated under reduced pressure. Purification by flash chromatography (pentane + 2 vol % NEt_3) yielded **5a** (151 mg, 75%, *E/Z* = 1:99) as a yellow oil.

^{1}H NMR ($CDCl_3$, 600 MHz) δ (ppm): 7.03–7.01 (m, 2H), 6.86–6.83 (m, 2H), 5.75–5.68 (m, 1H), 4.97–4.92 (m, 2H), 3.82 (s, 3H), 2.61 (d, *J* = 6.3 Hz, 2H), 2.32 (t, *J* = 7.1 Hz, 2H), 2.18 (q, *J* = 7.5 Hz, 2H), 1.31–1.20 (m, 8H), 1.05 (t, *J* = 7.5 Hz, 3H), 0.86 (m, *J* = 7.1 Hz, 3H).

^{13}C NMR ($CDCl_3$, 100 MHz) δ (ppm): 157.7, 137.8, 136.8, 136.0, 134.6, 129.7, 114.8, 113.2, 55.1, 37.1, 34.2, 31.8, 29.3, 28.4, 23.8, 22.6, 14.1, 13.4

IR (Diamond-ATR, neat) $\tilde{\nu}$ (cm^{-1}): 2956, 2926, 2872, 2856, 1636, 1608, 1508, 1458, 1442, 1374, 1286, 1242, 1174, 1104, 1038, 994, 908, 832, 810, 742, 704.

MS (70 eV, EI) *m/z* (%): 287 (15), 286 (M^+, 61), 257 (24), 202 (17), 201 (100), 187 (29), 184 (35), 174 (11), 173 (55), 172 (13), 161 (15), 160 (15), 159 (40), 158 (16), 147 (14), 145 (13), 128 (13), 121 (65), 115 (13), 91 (13), 57 (13), 55 (12), 43 (16), 43 (14), 41 (15).
HRMS (EI) for $\mathbf{C_{20}H_{30}O}$: (286.2297) 286.2290.

Synthesis of ethyl (2*E*)-2-allyl-3-(4-methoxyphenyl)non-2-enoate (5b)

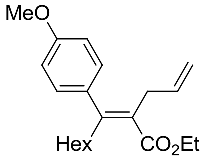

Prepared according to TP 2 from 2-(((1*E*)-1-allyl-2-(4-methoxyphenyl)oct-1-en-1-yl)thio)-2'-bromobiphenyl (**4a**, 436 mg, 1 mmol) and *s*-BuLi (1.35 mL, 1.1 mmol). After 10 min ethyl chloroformate (119 mg, 1.1 mmol) was added and the solution was stirred for 15 min. The reaction mixture was quenched with sat. NH_4Cl sol. (25 mL) and the resulting mixture was extracted with diethyl ether (3 × 50 mL). The combined organic layers were dried ($MgSO_4$), filtered, and concentrated under reduced pressure. Purification by flash chromatography (pentane + 2 vol % NEt_3) yielded **5b** (135 mg, 55%, *E/Z* = 95:5) as a colorless oil.
^{1}H NMR ($CDCl_3$, 400 MHz) δ (ppm): 7.10–7.07 (m, 2H), 6.95–6.92 (m, 2H), 5.76–5.66 (m, 1H), 4.98–4.90 (m, 2H), 4.15 (t, *J* = 7.0 Hz, 2H), 3.75 (s, 3H), 2.79 (d, *J* = 5.9 Hz, 2H), 1.22 (t, *J* = 7.1 Hz, 3H), 1.19–1.12 (m, 8H), 0.81–0.78 (m, 3H).
^{13}C NMR ($CDCl_3$, 100 MHz) δ (ppm): 169.1, 158.9, 148.7, 136.4, 132.9, 129.0, 127.6, 116.1, 114.1, 60.4, 55.5, 36.1, 35.5, 31.4, 28.9, 28.1, 22.4, 14.5, 14.3.
IR (Diamond-ATR, neat) $\tilde{\nu}$ (cm^{-1}): 2956, 2928, 2858, 1712, 1608, 1510, 1462, 1442, 1366, 1284, 1244, 1208, 1176, 1134, 1112, 1080, 1032, 1010, 994, 912, 834, 810, 752, 700, 668.
MS (70 eV, EI) *m/z* (%): 331 (18), 330 (M^+, 100), 329 (20), 285 (45), 260 (40), 257 (40), 245 (55), 227 (41), 214 (52), 199 (34), 199 (43), 187 (36), 186 (52), 185 (37), 173 (57), 172 (50), 171 (67), 159 (28), 158 (24), 147 (19), 145 (20), 134 (22), 128 (22), 121 (78), 108 (26).
HRMS (EI) for $\mathbf{C_{21}H_{30}O_3}$: (330.2195) 330.2179.

Synthesis of 1 Ethyl (4*E*)-4-allyl-5-(4-methoxyphenyl)-2-methyleneundec-4-enoate (5c)

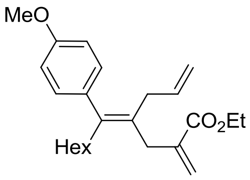

Prepared according to TP 2 from 2-(((1*E*)-1-allyl-2-(4-methoxyphenyl)oct-1-en-1-yl)thio)-2'-bromobiphenyl (**4a**, 436 mg, 1 mmol) and *s*-BuLi (1.35 mL, 1.1 mmol). After 10 min CuCN·2LiCl (1.1 mL, 1.1 mmol) was added and the resulting solution was stirred for 30 min. Then, ethyl 2-(bromomethyl)acrylate (452 mg, 1.5 mmol) was added and the mixture was stirred for 2 h warming up to 0 °C. The reaction mixture was quenched with sat. NH_4Cl sol. (25 mL) and the resulting mixture was extracted with diethyl ether (3 × 50 mL). The combined organic layers were dried ($MgSO_4$), filtered, and concentrated under reduced pressure. Purification by flash chromatography (pentane+ 2 vol % NEt_3) yielded **5c** (157 mg, 55%, *E/Z* = 99:1) as a yellow oil.

^{1}H NMR (C_6D_6, 600 MHz) *δ* (ppm): 7.12–7.10 (m, 2H), 6.87–6.84 (m, 2H), 6.46 (q, *J* = 1.7 Hz, 1H), 5.84–5.77 (m, 1H), 5.63 (q, *J* = 1.7 Hz, 1H), 5.08–5.03 (m, 2H), 4.09 (t, *J* = 7.1 Hz, 2H), 3.52 (t, *J* = 1.8 Hz, 2H), 3.38 (s, 3H), 2.78 (d, *J* = 6.3 Hz, 2H), 2.45–2.41 (m, 2H), 1.41–1.36 (m, 2H), 1.27–1.16 (m, 6H), 1.04 (t, *J*= 7.1 Hz, 3H), 0.87 (t, *J*= 7.1 Hz, 3H).

^{13}C NMR (C_6D_6, 150 MHz) *δ* (ppm): 166.8, 158.5, 140.9, 139.1, 137.3, 135.1, 129.5, 129.3, 128.0, 124.0, 115.4, 113.6, 60.4, 54.4, 37.8, 34.8, 32.8, 31.8, 29.3, 28.3, 22.7, 13.9.

IR (Diamond-ATR, neat) $\tilde{\nu}$ (cm^{-1}): 2927, 1716, 1608, 1510, 1464, 1283, 1243, 1175, 1134, 1034, 944, 833.

MS (70 eV, EI) *m/z* (%): 370 (M$^+$, 100), 285 (20), 257 (48), 239 (49), 211 (27), 185 (25), 173 (27), 172 (15), 171 (20), 159 (15), 147 (12), 122 (24), 121 (100), 59 (14), 43 (14), 41 (15).

HRMS (EI) for **$C_{24}H_{34}O_3$**: (370.2508) 370.2504.

Synthesis of 2'-bromo-[1,1'-biphenyl]-2-yl dimethylcarbamodithioate (13)

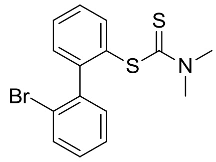

A: Synthesis of 2,2'-dibromo-1,1'-biphenyl

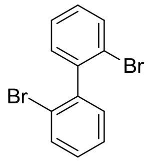

This compound was prepared from commercially available 1,2-dibromobenzene according to the procedure reported by Holmes et al. [4].

B: Synthesis of 2'-bromo-(1,1'-biphenyl)-2-yl dimethylcarbamodithioate (13)

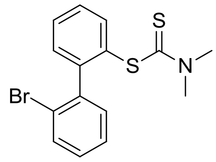

A dry and argon-flushed Schlenk flask, equipped with a magnetic stirring bar and a septum, was charged with 2,2'-dibromo-1,1'-biphenyl (7.8 g, 25 mmol) in THF (125 mL) and cooled to −78 °C. A solution of *n*-BuLi in hexanes (13.75 mL, 27.5 mmol) was added dropwise and the resulting mixture was stirred for 15 min. Then tetramethylthiuram disulfide (6.61 g, 27.5 mmol) was added in one portion and the suspension slowly warmed to 25 °C over 12 h. The reaction mixture was quenched with a sat. aq. NH_4Cl solution (100 mL), extracted with CH_2Cl_2 (3 × 200 mL) and dried over anhydrous $MgSO_4$. After filtration, the solvent was evaporated in vacuo. The crude product was purified by recrystallization from heptane:CH_2Cl_2 to give **13** (7.25 g, 82%) as colorless crystals.

m.p.: 150.7 – 152.3 °C.

^{1}H NMR ($CDCl_3$, 400 MHz) δ (ppm): 7.60 (dt, *J* = 7.7 Hz, 1.6 Hz, 2H), 7.54 (td, *J* = 7.5 Hz, 1.3 Hz, 1H), 7.48 (td, *J* = 7.6 Hz, 1.5 Hz, 1H), 7.42 (dd, *J* = 7.6 Hz, 1.6 Hz,

1H), 7.31 (dd, J = 7.7 Hz, 1.5 Hz, 1H), 7.27 (dd, J = 7.5 Hz, 1.1 Hz, 1H), 7.20 (dd, J = 7.7 Hz, 1.7 Hz, 1H), 3.42 (s, 3H), 3.25 (s, 3H).

^{13}C NMR (CDCl$_3$, 100 MHz) δ (ppm): 197.0, 146.4, 141.4, 138.4, 131.8, 131.1, 130.6, 130.2, 129.0, 128.7, 126.6, 123.6, 45.4, 42.1

IR (Diamond-ATR, neat) $\tilde{\nu}$ (cm^{-1}): 1496, 1454, 1431, 1374, 1243, 1144, 1055, 1025, 1004, 979, 944, 860, 764, 751, 720, 693, 655.

MS (70 eV, EI) *m/z* (%): 351 (M$^+$, 1, 272 (46), 184 (16), 152 (7), 139 (7), 88 (100), 73 (5), 43 (7).

HRMS (EI) for **$C_{15}H_{14}BrNS_2$**: (350.9751) 350.9733.

Synthesis of (Z)-(2'-bromo-[1,1'-biphenyl]-2-yl)(styryl)sulfane (12)

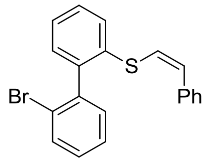

2’-Bromo-(1,1'-biphenyl)-2-yl dimethylcarbamodithioate (**13**, 7.05 g., 20 mmol) was added to a freshly prepared solution of NaOEt in EtOH, made from sodium (2.37 g, 25 mmol) and absolute ethanol (25 mL). Freshly distilled phenylacetylene (3.0 g., 30 mmol) was then added and, after 15 hours of heating under reflux, the resulting solution was poured into water (100 mL), extracted with CH_2Cl_2 (3 × 200 mL) and dried over anhydrous $MgSO_4$. After filtration, the solvent was evaporated in vacuo. Purification by flash chromatography (pentane + 1 vol % NEt_3) yielded **12** (157 mg, 74%, *E/Z* > 1:99) as a yellowish oil.

^{1}H NMR (CDCl$_3$, 300 MHz) δ (ppm): 7.71 (d, J = 8.0 Hz, 1H), 7.62 (d, J = 7.5 Hz, 1H), 7.50–7.46 (m, 2H), 7.43 (dd, J = 5.7 Hz, 1.5 Hz, 1H), 7.40–7.37 (m, 2H), 7.35 (d, J = 6.1 Hz, 2H), 7.32–7.22 (m, 4H), 6.59 (d, J = 10.5 Hz, 1H), 6.48 (d, J = 10.5 Hz, 1H).

^{13}C NMR (CDCl$_3$, 75 MHz) δ (ppm): 142.2, 141.2, 136.3, 135.8, 132.6, 131.3, 130.9, 130.3, 129.2, 128.9, 128.7, 128.1, 127.9, 127.1, 127.0, 127.0, 125.9, 123.8.

IR (Diamond-ATR, neat) $\tilde{\nu}$ (cm^{-1}): 3050, 3019, 1594, 1562, 1490, 1455, 1422, 1354, 1082, 1055, 1027, 1003, 944, 943, 909, 846, 750, 725, 689, 659.

MS (70 eV, EI) *m/z* (%): 366 (M$^+$, 7), 287 (100), 209 (16), 184 (39), 52 (16), 139 (11), 103 (55), 77 (22), 43 (43).

HRMS (EI) for **$C_{20}H_{15}BrS$**: (366.0078) 366.0075.

Synthesis of (*Z*)-1-styrylcyclopentanol (15a)

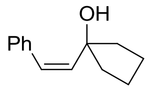

Prepared according to TP 2 from (*Z*)-(2'-bromo-[1,1'-biphenyl]-2-yl)(styryl)sulfane (**12**, 367 mg, 1 mmol) and *t*-BuLi (1.6 mL, 1.6 mmol). After 10 min cyclopentanone (67 mg, 0.8 mmol) was added and the resulting solution was stirred for 15 min. Then, the reaction mixture was quenched with sat. $NaHCO_3$ sol. (25 mL) and the resulting mixture was extracted with diethyl ether (3 × 50 mL). The combined organic layers were dried ($MgSO_4$), filtered, and concentrated under reduced pressure. Purification by flash chromatography (aluminium oxide, pentane) yielded **15a** (107 mg, 71%, *E/Z* > 1:99) as a yellow oil.

^{1}H NMR (DMSO-d6, 400 MHz) δ (ppm): 7.53 (d, *J* = 7.4 Hz, 2H), 7.27 (t, *J* = 7.5 Hz, 2H), 7.18 (t, *J* = 7.3 Hz, 1H), 6.33 (d, *J* = 12.7 Hz, 1H), 5.80 (d, *J* = 12.7 Hz, 1 H), 4.59–4.46 (s, 1H), 1.77–1.45 (m, 8 H)

^{13}C NMR (DMSO-d6, 100 MHz) δ (ppm): 138.6, 137.3, 129.6, 128.8, 127.4, 126.5, 79.4, 40.6, 23.1.

IR (Diamond-ATR, neat) $\tilde{\nu}$ (cm^{-1}): 2953, 2870, 1492, 1447, 1183, 1071, 1028, 991, 945, 915, 886, 840, 767, 694.

MS (70 eV, EI) *m/z* (%): 188 (M^+, 3), 145 (3), 105 (3), 91 (7), 88 (5), 70 (10), 61 (14), 45 (13), 43 (100).

HRMS (EI) for **$C_{13}H_{16}O$**: (188.1201) 188.1193.

Synthesis of (*Z*)-1,1,1-trifluoro-2,4-diphenylbut-3-en-2-ol (15b)

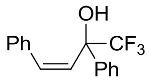

Prepared according to TP 2 from (*Z*)-(2'-bromo-[1,1'-biphenyl]-2-yl)(styryl)sulfane (**12**, 367 mg, 1 mmol) and *t*-BuLi (1.6 mL, 1.6 mmol). After 10 min α,α,α-trifluoroacetophenone (139 mg, 0.8 mmol) was added and the resulting solution was stirred for 15 min. Then, the reaction mixture was quenched with sat. $NaHCO_3$ sol. (25 mL) and the resulting mixture was extracted with diethyl ether (3 × 50 mL). The combined organic layers were dried ($MgSO_4$), filtered, and concentrated under reduced pressure. Purification by flash chromatography (aluminum oxide, pentane) yielded **15b** (183 mg, 82%, *E/Z* > 1:99) as a yellow oil.

^{1}H NMR (DMSO-d6, 400 MHz) *δ* (ppm): 7.54 (d, *J* = 7.0 Hz, 2H), 7.28–7.23 (m, 4H), 7.16–7.12 (s, 1H), 7.08–7.02 (m, 3H), 6.82 (d, *J* = 12.9 Hz, 1H), 6.29 (d, *J* = 12.9 Hz, 1H), 3.31 (s, 1H)

^{13}C NMR (DMSO-d6, 100 MHz) *δ* (ppm): 137.8, 135.8, 135.2, 130.4, 128.5, 128.0, 127.9, 127.8, 127.7, 127.6, 126.3 (q, *J* = 288.2 Hz), 75.7 (q, *J* = 28.6 Hz).

IR (Diamond-ATR, neat) $\tilde{\nu}$ (cm^{-1}): 1494, 1449, 1277, 1253, 1183, 1151, 1125, 1071, 1030, 986, 946, 911, 761, 730, 694.

MS (70 eV, EI) *m/z* (%): 278 (M^+, 3), 209 (39), 131 (16), 105 (10), 103 (13), 77 (17), 61 (9), 45 (11), 43 (100).

HRMS (EI) for **$C_{16}H_{13}F_3O$**: (278.0918) 278.0916.

References

1. Dunst, C.; Metzger, A.; Zaburdaeva, E. A.; Knochel, P. *Synthesis* **2011,** 3453. doi:10.1055/s-0030-1260210
2. Wilson, H. F.; Tarbell, D. S. *J. Am. Chem. Soc.* **1950,** *72,* 5200. doi:10.1021/ja01167a117
3. Bernhardt, S.; Manolikakes, G.; Kunz, T.; Knochel, P. *Angew. Chem.* **2010,** *122,* 4769; doi: 10.1002/ange.201000634; *Angew. Chem. Int. Ed.* **2010,** *49,* 4665. doi: 10.1002/anie.201000634
4. Chan, K. L.; Watkins, S. E.; Mak, C. S. K.; McKiernan, M. J.; Towns, C. R.; Pascua, S. I.; Holmes, A. B. *Chem. Commun.* **2005,** 5766. doi:10.1039/b511208g
